# Supplementary material for: Experiences of community reintegration after obstetric fistula repair at Jean Paul 2 hospital, Conakry, Guinea
Source: PLOS Glob Public Health. 2024 Aug 6;4(8):e0003581. doi: 10.1371/journal.pgph.0003581 (PMC11302855; doi:10.1371/journal.pgph.0003581)
Supplement: S1 Checklist — (DOCX) [file pgph.0003581.s001.docx]

| COREQ) : List 32-point inspection | | |
| --- | --- | --- |
| **Domain 1: Team of Research and Reflexivity** |  |  |
| 1. Interviewer/Facilitator | What individual(s) led the interview or discussion group? | Hady Kaba (interviewer) |
| 2. References | What are the qualifications of the researcher? For example, doctorate, MD, MSc. | MD |
| 3. Occupancy | What was their occupation at the time of the study? | Student |
| 4. The Gender | Was the researcher male or female? | Male |
| 5. Experience and Training | What was the experience or training of the researcher? | Epidemiology |
| Relationships with the Participants |  |  |
| 6. Establishment of a Relationship | Was a relationship established before the study began? | No |
| 7. Awareness of the Investigator by the Participant | What did the participants know about the researcher? For example, their personal goals, reasons for conducting the research. | No knowledge |
| 8. Features of the Investigator | What features did the interviewer/host possess that were reported? For example, biases, hypotheses, interests in the research topic. | Interested in gender issues and maternal and child health |
| **Domain 2: Design of the Study** |  |  |
| **Theoretical Framework** |  |  |
| 9. Methodological Orientation and Theory | What is the stated methodological focus supporting the study? | Thematic analysis |
| **Selection of the Participants** |  |  |
| 10. Sampling | How were the participants selected? For example, purposive, convenience, consecutive, snowball. | Convenience |
| 11. Method Approach | How were the participants approached? For example, face-to-face, by phone, by mail, by email. | By telephone |
| 12. Size of the Sample | How many participants were in the study? | 10 participants |
| 13. Non-Participation | How many people refused to participate or dropped out? For what reasons? | 15 non-participants due to lack of response during the solicitation phase |
| **Settings** |  |  |
| 14. Data Collection Setting | Where was the data collected? For example, at home, in a clinical setting, at the workplace. | At the Jean Paul 2 hospital - Conakry |
| 15. Presence of Non-Participants | Were there other people besides the participants and researchers? | No |
| 16. Sample Description | What are the important features of the sample? For example, demographic data, dates, etc. | Average age of participants 32.4 years, living in rural areas, no education and newborn death. |
| **Data Collection** |  |  |
| 17. Guide Maintenance | Did the authors provide questions, prompts, or guides? Was the project piloted? | No |
| 18. Repetition of the Interviews | Were repeated interviews conducted? If yes, how many? | No |
| 19. Audio/Visual Recording | Did the research use audio or visual recordings to collect data? | Yes |
| 20. Ground Notes | Were field notes taken during and/or after the interview or discussion? | Field notes were taken at the time of the interview |
| 21. Duration of Interviews | What was the duration of the interviews or discussions? | Average duration of interviews: 30 min – 60 min |
| 22. Data Saturation | Was data saturation achieved? | Yes |
| 23. Feedback of Notes | Did the transcriptions return to participants for comments or corrections? | No |
| **Domain 3: Analysis and Conclusions** |  |  |
| **Analysis of the Data** |  |  |
| 24. Number of Coders | How many coders coded the data? | 2 persons |
| 25. Description of the Tree Coding | Did the authors provide a description of the coding tree? | No |
| 26. Derivation of the Themes | Were the themes identified in advance or derived from the data? | Themes were identified in advance |
| 27. Software | What software, if applicable, was used to manage data? | Nvivo12 software |
| 28. Participant Feedback | Did participants give feedback on the results? | No |
| **Reports** |  |  |
| 29. Presented Quotes | Were quotes from participants presented to illustrate themes/results? Were the quotes identified? | Yes |
| 30. Consistency of Data and Conclusions | Is there consistency between the presented data and conclusions? | Yes |
| 31. Clarity of Main Themes | Were the main themes clearly presented in the conclusions? | Yes |
| 32. Clarity of Minor Themes | Was there a discussion of minor themes? | No |
