# Supplementary material for: Experiences of community reintegration after obstetric fistula repair at Jean Paul 2 hospital, Conakry, Guinea
Source: PLOS Glob Public Health. 2024 Aug 6;4(8):e0003581. doi: 10.1371/journal.pgph.0003581 (PMC11302855; doi:10.1371/journal.pgph.0003581)
Supplement: S1 Text — (DOCX) [file pgph.0003581.s002.docx]

**INTERVIEW GUIDE**

# Presentation

My name _______________in collaboration with the Jean Paul 2 hospital, we are conducting a

study on obstetric fistulas. You have been asked, with your consent, to participate in this study. Our aim is to understand the psychological and social consequences of obstetric fistula, to better orient the overall care of those affected according to their needs. Your contribution will help us to provide knowledge that will enable decision-makers to develop more effective and efficient programs for women suffering from fistula.

# Socio-demographic data of the respondent

Last name: __________________ First name: _____________________

Age: /_ /

Marital status: Married /_ / Divorced /_ / Single /_ /

Occupation: / /

Level of education: None /_ / Primary /_ / Secondary /_ / Higher education /_/

| THEMES | GENERAL QUESTIONS | FOLLOW-UP QUESTIONS |
| --- | --- | --- |
| PAST  EXPERIENCES | Can you tell me about your experience with the disease? How do you feel about how your family, community and friends have viewed you since your return from hospital? |  |
| SOCIAL SUPPORT  EXPERIENCES | Would you like to tell me about the help you've received since returning to your family and community?  Apart from family and members of your community, do you think you have benefited from other means that have helped you within the community? | What do you mean? |
|  |  | Can you tell me more about it? |
| REINSERTION EXPERIENCE | Would you like to tell me how you participate in social  activities (e.g. ceremonies, family discussions and decisions)? What do you think should be done to help women with fistula when they return to live in the community? |  |
