# Supplementary material for: Experiences of community reintegration after obstetric fistula repair at Jean Paul 2 hospital, Conakry, Guinea
Source: PLOS Glob Public Health. 2024 Aug 6;4(8):e0003581. doi: 10.1371/journal.pgph.0003581 (PMC11302855; doi:10.1371/journal.pgph.0003581)
Supplement: S2 Text — (DOCX) [file pgph.0003581.s003.docx]

**MEDICAL AND OBSTETRICAL HISTORY**

- - How many times have you been pregnant? \ \
  - How many times have you given birth? \ \
  - Number of living children? \ \
  - Where did you give birth**?**
    - Home \ _\
    - Health Center \ _\
    - Hospital \ _\
  - Did you receive assistance during the birth that caused the obstetric fistula?
    - Yes **\** \
    - No \ \
  - How did you give birth?
    - Low track \ _\
    - Cesarean section \ _\
  - What was the newborn's outcome at birth?
    - Vivant \ _\
    - Deceased \ _\
  - What is the birth rank?
  - What is the number of years of life with obstetric fistula? \ \
  - What was the nature of the obstetric fistula?
- VVF (vesico-vaginal fistula) \ \
- RVF (recto-vaginal fistula) \ \
- MIXED \ \
  - How many times have you been operated on since this fistula appeared? \_\
