# Supplementary material for: Experiences of community reintegration after obstetric fistula repair at Jean Paul 2 hospital, Conakry, Guinea
Source: PLOS Glob Public Health. 2024 Aug 6;4(8):e0003581. doi: 10.1371/journal.pgph.0003581 (PMC11302855; doi:10.1371/journal.pgph.0003581)
Supplement: Attachment — Submitted filename: renamed_4edae.docx [file pgph.0003581.s004.docx]

Bonjour Dre Gupta,

Merci beaucoup à vous et aux reviseurs pour vos commentaires et suggestions, qui ont permis de perfectionner le manuscrit. Nous vous prions de trouver ici les réponses aux questions soulevées.

| COMMENTAIRE | RÉPONSE |
| --- | --- |
| 1. Veuillez noter que nous recommandons aux auteurs d'utiliser la liste de contrôle du COREQ ou d'autres listes de contrôle pertinentes répertoriées par le Réseau de l'Équateur, telles que le SRQR, afin d'assurer l'exhaustivité des rapports. ([http://journals.plos.org/globalpublichealth/s/submission-guidelines#loc-qualitative-resea rch](http://journals.plos.org/globalpublichealth/s/submission-guidelines#loc-qualitative-resea%20rch)). Veuillez inclure ces informations dans la section des méthodes et modifier le contenu pour vous assurer que les critères sont remplis. | La liste COREQ a été complétée pour répondre à cette première interrogation |
| 2. Veuillez préciser dans le texte si les noms inclus avec les citations sont les noms de participants ou des pseudonymes et si le consentement du CER a été obtenu pour ce type de rapport. | Dans les citations, ce sont des pseudonymes que nous avons utilisé dans le souci de préserver l’anonymat des participants avec un consentement éclairé et signé par les participants |
| 3. À l'heure actuelle, les résultats sont présentés dans la section Méthodes. Je recommanderais de déplacer la section des résultats pour plus de clarté. | Les résultats ont été séparés de la partie méthodologie |
| 4. Le titre de cette étude indique qu'il s'agit d'une « étude de cas », ce qui n'est pas justifié dans la section sur les méthodes. Veuillez envisager de changer le titre de l'article ou de justifier la méthodologie de l'étude de cas. | Le nouveau titre envisagé : Experiences of community reintegration after obstetric fistula repair at Jean Paul 2 hospital, Conakry, Guinea. |
| 5. Veuillez remplir tous les éléments de la liste de contrôle de la recherche sur les participants humains qui sont pertinents pour votre soumission en suivant ce lien : <http://journals.plos.org/plosone/s/file?id=dc11/PLOSOne_Human_Subjects_Research_Checklist.docx>. | La liste de contrôle de la recherche sur les participants humains a été complétée |

Nous vous remercions encore pour les commentaires constructifs et attendons avec impatience votre réponse.

Cordialement

Dr Hady Kaba
