# Supplementary material for: Experiences of community reintegration after obstetric fistula repair at Jean Paul 2 hospital, Conakry, Guinea
Source: PLOS Glob Public Health. 2024 Aug 6;4(8):e0003581. doi: 10.1371/journal.pgph.0003581 (PMC11302855; doi:10.1371/journal.pgph.0003581)
Supplement: Attachment — Submitted filename: Response to reviewers.docx [file pgph.0003581.s005.docx]

Hello Dr. Gupta,

Many thanks to you and to the reviewers for your comments and suggestions, which have helped to improve the manuscript. Please find here the answers to the questions raised.

| COMMENT | RESPONSE |
| --- | --- |
| 1. Please note that we recommend that authors use the COREQ checklist or other relevant checklists listed by the Equator Network, such as SRQR, to ensure the completeness of reports. ([http://journals.plos.org/globalpublichealt](http://journals.plos.org/globalpublichealth/s/submission-guidelines#loc-qualitative-resea%20rch) [h/s/submission-guidelines#loc-qualitative-](http://journals.plos.org/globalpublichealth/s/submission-guidelines#loc-qualitative-resea%20rch) [resea rch](http://journals.plos.org/globalpublichealth/s/submission-guidelines#loc-qualitative-resea%20rch)). Please include this information in the methods section and edit the content to ensure the criteria are met. | The COREQ list has been completed to answer this first question. |
| 2. Please specify in the text whether the names included with the quotations are participants' names or pseudonyms and whether the REC's consent has been obtained for this type of report. | In the quotations, we have used pseudonyms in order to preserve the anonymity of the participants, with informed consent signed by the participants. |
| 3. At present, the results are presented in the Methods section. I would recommend moving the results section for greater clarity. | The results have been separated from the methodology section |
| 4. The title of this study indicates that it is a "case study", which is not justified in the methods section. Please consider changing the title of the article or justifying the case study methodology. | New title considered: Experiences of community reintegration after obstetric fistula repair at Jean Paul 2 hospital, Conakry, Guinea. |
| 5. Please complete all items on the Human Participant Research Checklist that are relevant to your submission by following this link: <http://journals.plos.org/plosone/s/file?id=d> c11/PLOSOne_Human_Subjects_Researc h_Checklist.docx. | The checklist for research involving human participants has been completed. |

We thank you again for the constructive feedback and look forward to your reply. Sincerely

Dr Hady Kaba
