# Supplementary material for: Experiences of community reintegration after obstetric fistula repair at Jean Paul 2 hospital, Conakry, Guinea
Source: PLOS Glob Public Health. 2024 Aug 6;4(8):e0003581. doi: 10.1371/journal.pgph.0003581 (PMC11302855; doi:10.1371/journal.pgph.0003581)
Supplement: Attachment — Submitted filename: Response to Reviewers.docx [file pgph.0003581.s006.docx]

**Subject: Elements of clarification to the comments of the evaluator of my article**

Dear reviewer,

I sincerely thank you for taking the time to review my article and for your constructive comments. Your expertise and suggestions are extremely valuable to me.

Concerning the questionnaire used in the study, I attach in annex the socio-demographic questionnaire as well as the interview guide which were used on page 22-23 of the manuscript.

Regarding the study period (2017-2020), from the start of the project, we collaborated with hospital authorities to obtain recent and accessible data. Additional details on this have been included in the manuscript on page 5:

" The Jean Paul 2 Hospital, located in the commune of Ratoma in Guinea, is a primary and secondary health care establishment placed under the supervision of the Ministry of Social Affairs. This hospital houses a center specializing in the care and rehabilitation of women who have undergone obstetric fistula repairs. The center has a maternity service with a unit dedicated specifically to women victims of fistula, offering them care and appropriate medical monitoring. The medical team is made up of three obstetrician-gynecologists, two surgeons specializing in fistulas, as well as midwives and social workers."

Your suggestion to include information on the type of health facility (primary, secondary or tertiary) is particularly relevant. The hospital in question offers primary and secondary care, with a center specializing in the management of fistulas. This information has been supported and added to the manuscript on page 5:

" To analyze the community integration of women victims of obstetric fistula after treatment at the Jean Paul 2 Hospital, we chose the period 2017-2020, after consultation with the hospital authorities. This period was chosen for the relevance and availability of recent data. Previous years presented limitations in terms of traceability and availability of data, mainly due to the absence of a computerized patient record management system, with information being recorded in handwritten registers, making their management difficult."

I have also corrected the spelling error regarding the term "RECOMMENDATIONS" on page 18.

I hope these answers address your concerns. Thank you again for your detailed evaluation and for your contribution to improving my work. I remain at your disposal for any questions or requests for additional clarification.

Best regards,

Hady Kaba

Ps: Please find below the interview guide and the socio-demographic questionnaire

**INTERVIEW GUIDE**

# Presentation

My name _______________in collaboration with the Jean Paul 2 hospital, we are conducting a study on obstetric fistulas. You have been asked, with your consent, to participate in this study. Our aim is to understand the psychological and social consequences of obstetric fistula, to better orient the overall care of those affected according to their needs. Your contribution will help us to provide knowledge that will enable decision-makers to develop more effective and efficient programs for women suffering from fistula.

# Socio-demographic data of the respondent

Last name: __________________	First name: _____________________

Age: /_ /

Marital status: Married	/_ /	Divorced	/_ /	Single /_ /

Occupation: /	/

Level of education: None /_ /	Primary /_ /	Secondary	/_ /	Higher education /_/

| THEMES | GENERAL QUESTIONS | FOLLOW-UP QUESTIONS |
| --- | --- | --- |
| PAST  EXPERIENCES | Can you tell me about your experience with the disease? How do you feel about how your family, community and friends have viewed you since your return from hospital? |  |
| SOCIAL SUPPORT  EXPERIENCES | Would you like to tell me about the help you've received since returning to your family and community?  Apart from family and members of your community, do you think you have benefited from other means that have helped you within the community? | What do you mean? |
|  |  | Can you tell me more about it? |
| REINSERTION EXPERIENCE | Would you like to tell me how you participate in social  activities (e.g. ceremonies, family discussions and decisions)? What do you think should be done to help women with fistula when they return to live in the community? |  |

**MEDICAL AND OBSTETRICAL HISTORY**

1. **How many times have you been pregnant?** ____
2. **How many times have you given birth?** ____
3. **Number of living children?** ____
4. **Where did you give birth?**
   - Home: ___
   - Health Center: ___
   - Hospital: ___
5. **Did you receive assistance during the birth that caused the obstetric fistula?**
   - Yes: ___
   - No: ___
6. **How did you give birth?**
   - Low track: ___
   - Cesarean section: ___
7. **What was the newborn's outcome at birth?**
   - Alive: ___
   - Deceased: ___
8. **What is the birth rank?** ____
9. **What is the number of years of life with obstetric fistula?** ____
10. **What was the nature of the obstetric fistula?**
    - VVF (vesico-vaginal fistula): ___
    - RVF (recto-vaginal fistula): ___
    - MIXED: ___
11. **How many times have you been operated on since this fistula appeared?** ____
